# Toxicity of Selected Acaricides to Honey Bees (*Apis mellifera*) and Varroa (*Varroa destructor* Anderson and Trueman) and Their Use in Controlling Varroa within Honey Bee Colonies

**DOI:** 10.3390/insects9020055

**Published:** 2018-05-10

**Authors:** Aleš Gregorc, Mohamed Alburaki, Blair Sampson, Patricia R. Knight, John Adamczyk

**Affiliations:** 1 Center for Costal Horticulture Research, Mississippi State University, Poplarville, MS 39470, USA; prk3@msstate.edu; 2 Agricultural Institute of Slovenia, 1000 Ljubljana, Slovenia and Faculty of Agriculture and Life Sciences, University of Maribor, 2000 Maribor, Slovenia; 3 Department of Biological Sciences, The University of Southern Mississippi, Hattiesburg, MS 39406, USA; Mohamed.Alburaki@usm.edu; 4 USDA ARS, Thad Cochran Southern Horticultural Research Laboratory, Poplarville, MS 39470, USA; Blair.Sampson@ARS.USDA.GOV (B.S.); John.Adamczyk@ARS.USDA.GOV (J.A.)

**Keywords:** *Varroa destructor*, honey bee, caged-bees, varroacides

## Abstract

The efficacies of various acaricides in order to control a parasitic mite, the Varroa mite, *Varroa destructor*, of honey bees, were measured in two different settings, namely, in laboratory caged honey bees and in queen-right honey bee colonies. The Varroa infestation levels before, during, and after the acaricide treatments were determined in two ways, namely: (1) using the sugar shake protocol to count mites on bees and (2) directly counting the dead mites on the hive bottom inserts. The acaricides that were evaluated were coumaphos, tau-fluvalinate, amitraz, thymol, and natural plant compounds (hop acids), which were the active ingredients. The acaricide efficacies in the colonies were evaluated in conjunction with the final coumaphos applications. All of the tested acaricides significantly increased the overall Varroa mortality in the laboratory experiment. Their highest efficiencies were recorded at 6 h post-treatment, except for coumaphos and thymol, which exhibited longer and more consistent activity. In the honey bee colonies, a higher Varroa mortality was recorded in all of the treatments, compared with the natural Varroa mortality during the pretreatment period. The acaricide toxicity to the Varroa mites was consistent in both the caged adult honey bees and workers in the queen-right colonies, although, two of these acaricides, coumaphos at the highest doses and hop acids, were comparatively more toxic to the worker bees.

## 1. Introduction

*Varroa destructor* [1] is a worldwide parasite of *Apis*, which causes significant brood and adult mortality in colonies of European honey bees (*Apis mellifera* L.). Reliable Varroa population diagnosis, monitoring, and control are the prominent issues in modern beekeeping, which, if improved, could reduce or reverse the global losses of honey bees and their colonies [2]. If the Varroa mites are left untreated, the commercial colonies will normally die within three to five years. Worsening the situation are the populations of the Varroa mites that have evolved a resistance to many of the synthetic acaricides [3,4,5]. Therefore, beekeepers increasingly rely on acaricides with different modes of action, many of which contain essential oils and organic acids as active ingredients [5,6,7].

A wide array of chemotherapeutic, api-technical, biological, and behavioral methods are available for the control of the Varroa mites within honey bee colonies [2]. To minimize the pesticide residues in the colonies and hive products, and to thwart the acaricide resistance [8,9], beekeepers increasingly rely on organic oils and acids, which are variably effective controls of Varroa. Many beekeepers prefer to apply synthetic pyrethroids, such as tau-fluvalinate (Apistan^®^) and flumethrin (Bayvarol^®^), as well as the organophosphate, coumaphos (CheckMite^®^), and the octopamine analog, amitraz (Apivar^®^), because of their low cost, ease of application, prolonged activity, and perceived high efficacy [10].

However, an over-reliance on these products, without adequate rotation, is associated with increasing incidences of Varroa resistance, particularly to the pyrethroids [11,12,13,14,15]. Botanical-based acaricides are also commercially registered for Varroa control. The first of these is Apiguard^®^ (Vita Europe Ltd.; Basingstoke, UK), which contains the volatile monoterpenoid, thymol, an active ingredient that is gradually released within bee colonies from food-grade gel packets [16,17,18]. Experimentally, Apiguard^®^ is shown to induce 76% to 95% of the mortality in Varroa mites [4,19]. In the fully developed colonies containing a reproductive queen, workers capped brood, and honey stores in combs that are maintained in a continental climate, Apiguard^®^ kills less than 50% of the mites [20]. HopGuard^®^, is another botanical-based acaricide with beta hop acids as active ingredients. HopGuard is effective against Varroa within colonies, whether the frames contain the open or sealed brood. HopGuard^®^ kills >80% of Varroa in both the large and small honey bee colonies containing brood, with 65% of the mites dying within the first 24 h post-treatment [21]. However, HopGuard becomes a less effective mite treatment during the peak brood production [22].

The first objective of this study was to determine, in the laboratory, the comparative toxicities of five acaricidal products (Apistan^®^, Apivar^®^, Apiguard^®^, HopGuard^®^, and CheckMite^®^) to both the Varroa mites and host bees. The second objective was to test the acaricidal efficacies of these same five products as a first hive treatment, followed by a second CheckMite^®^ application to the queen-right colonies in the field. The accuracy of the Varroa populations, using the sugar shake method, was tested by comparing the sugar-shake counts with those of an established protocol, which involved tallying the dead mites atop the hive bottom inserts.

## 2. Materials and Methods

The laboratory experiments tested the efficacy of the five acaricidal products (Apistan^®^, Apivar^®^, Apiguard^®^, HopGuard^®^, and CheckMite^®^) in order to kill the Varroa mites and to determine the product toxicities to the worker honey bees. The products that were widely used in beekeeping practice were also tested for Varroa suppression under field conditions, using fully developed queen-right honey bee colonies. Estimates of the Varroa infestation in the field colonies were derived using two methods, namely: (1) an established protocol that involved counting the dead mites on the hive bottom inserts and (2) determining the adult bees’ infestation using the sugar shake method. 

### 2.1. Laboratory Toxicity Experiment

**Figure 1:**
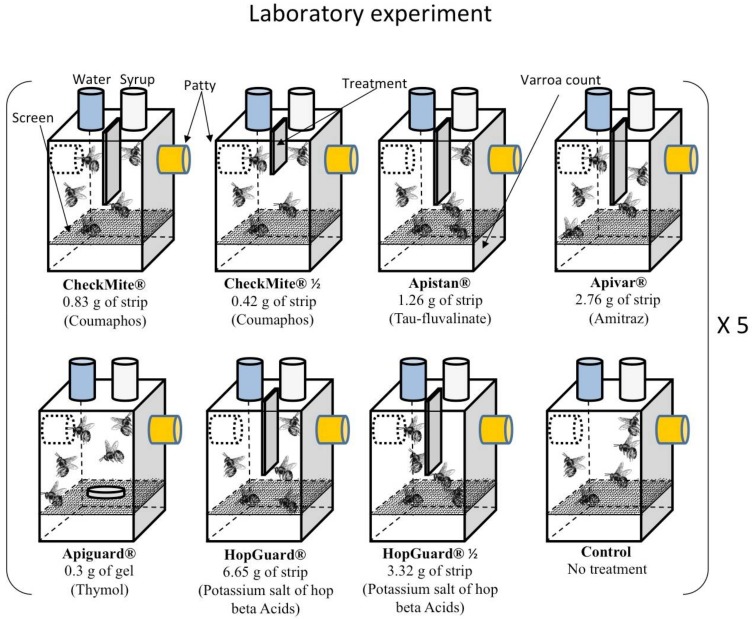
Experimental design and the application rates of commercial acaricides applied in each laboratory cage. The control treatment received no acaricidal treatment. Caged bees were provided with sugar syrup, water, and pollen patty. Five replicates were carried out for each treatment, for a total of 40 cages.

In the laboratory studies, the bees were kept in 11.4 × 6.3 × 15.2 cm (W:D:H) cages, each with a top, bottom, and sides made from clear acrylic, and a front, back, and bottom that was constructed from a 2.5 mm wire screen mesh. The mesh bottom insert was placed approximately 2.5 cm above the cage bottom, through which the dead Varroa mites could fall onto the cage bottom for later collection and counting. The cages were equipped with two 60 mL polypropylene bottles (United States Plastic Crop^®^, Lima, OH, USA), each with a lid containing three small holes (Figure 1). One bottle was filled with a (1:1) sugar in water solution and the other with water. Each bottle was placed upside down in two of the holes that were drilled in the cage top, which allowed the bees to feed from those bottles. The cage had a hole drilled through each side, where the rubber plug pollen feeders (Sigma-Aldrich^®^, St. Louis, MO, USA) were placed. Lastly, two more small holes were drilled in the acrylic top between the sugar and water feeder holes, and were used to hang the acaricidal product with the wire inside the cage.

Eight different treatment groups were established; seven separate treatment groups and a non-treated control group. Each group consisted of five replicated cages. The caged worker bees of each treatment group received a specific acaricidal dose, and cages of the control group received no treatment (Figure 1). The eight groups (seven treatment and one control) consisted of the following: a (1) coumaphos treatment group with 0.83 g of a full CheckMite^®^ strip (CheckMite^®^ 1) (CAS # KPOB5KD, Shawnee Mission, KS, USA); (2) coumaphos treatment group with a half dose, 0.42 g of a full CheckMite^®^ strip (CheckMite^®^ ½); (3) Tau-fluvalinate treatment group: 1.26 g (50 × 30 mm) of a full Apistan^®^ strip, (CAS # 102851-06-9, Wellmark International, IL, USA); (4) amitraz treatment group: 2.76 g (38 × 40 mm) of a full Apivar^®^ strip (CAS # 87243-1, Veto-pharma, New York, NY, USA); (5) thymol treatment group: 0.3 g of Apiguard^®^ (CAS # 79671-1, Vita Europe Limited, Valdosta, GA, USA); (6) natural plant compounds (hop acids) treatment group: 6.65 g (70 × 33 mm) of full HopGuard^®^ cardboard strip (HopGuard^®^ 1); (7) HopGuard^®^ treatment group: 3.32 g (35 × 33 mm) of full HopGuard^®^ cardboard strip (HopGuard^®^ ½) with the potassium salt of the hop beta acids as the active ingredient (CAS # DC301120716Y30816, Betatec, Mann Lake Ltd., Hackensack, MN, USA); and (8) a control group, which received no acaricidal treatment.

The worker bees were obtained from three Varroa-infested colonies from our current stock, which was headed by Italian-bred queens, *Apis mellifera ligustica*. The bees were brushed from the brood frames into a clean bucket and then randomly assigned to cages at a density of ~250 bees per cage. The cages were maintained in an unlit incubator set at a near constant 28 °C and 65% relative humidity values (RH). The caged bees were provided with (1:1) sugar syrup, water, and pollen patty, *ad libitum*, using the methods described above. 

Pieces of each original acaricidal product were fixed with a wire and hung from the inside of the cage top, which ensured exposure to the bees from both sides, and the amount of Apiguard^®^ was placed in a small plastic lid on the bottom mesh insert, so as to expose and ensure contact with all of the bees within the cage. The treatment dose of Apiguard that was applied to the caged bees was calculated according to the total number of bees per cage (250), by extrapolating the data from the treated colonies in a standard Langstroth (LR) hive that received a full dose for a standard developed honey bee colony, as was advised by the manufacturer. Similar to what was done in the full-sized colonies, the acaricidal products were cut to a size that ensured that the caged-bees with Varroa contacted the exposed part of the strip and received an effective acaricidal dose. 

The control group without the acaricidal treatment received the same food as the treated bees (i.e., sugar solution, water, and pollen patty). The acaricidal treatments that were administered to the caged-bees were meant to mimic those that were applied to the queen-right honeybee colonies, with the additional advantage of simultaneously testing the toxicities on both the mites and host bees. During the experiment, any mites that were killed and dislodged by the treatment, fell through the screen insert onto the bottom of the cage and were then collected and counted at 6, 24, and 48 h intervals. The dead bees were then collected from above the bottom screen. The counts of the dead Varroa and bees that exceeded the counts in the control cages were considered to have been killed by the specific acaricidal treatment. All of the Varroa that remained on the adult bees throughout the test period were considered to be tolerant of or resistant to the treatment. At the end of the experiment (48 h), all of the cages with the remaining bees were placed in a freezer at −20 °C. Once all of the bees in a cage were euthanized, they were rinsed thoroughly through a strainer and counted along with their total mite load (mites per cage).

### 2.2. Field Experiment

**Figure 2:**
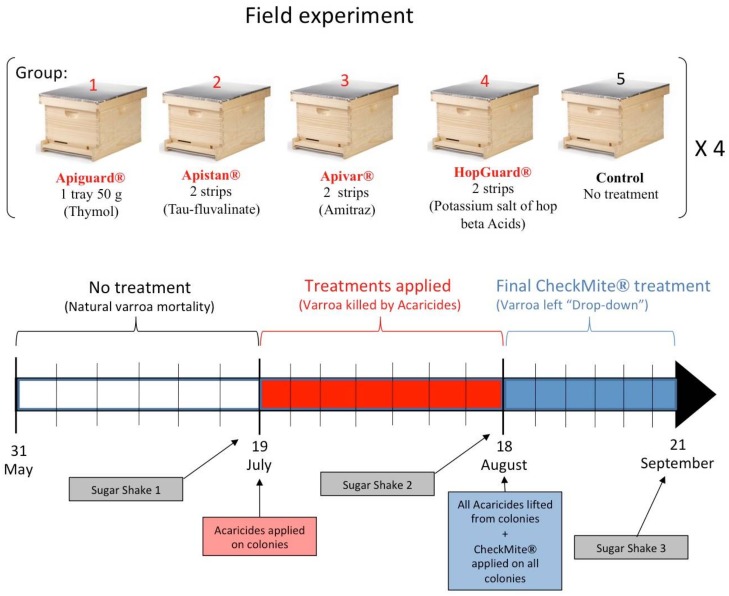
Field experimental design showing the acaricidal products that were tested, amounts of product used in each honey bee colony, and the timeline detailing the products and their application dates.

Using the same acaricidal products as those from the previous laboratory experiment, 20 honey bee colonies were used to test the Varroa control in the field. All of the colonies that were founded by Italian queens, *Apis mellifera ligustica*, were established in autumn 2016, at an apiary that was located at the Mississippi State University’s Subunit in McNeill, MS (30°39′46″ N, 89°38′01″ W). The experimental colonies were queen-right, fully developed, and housed in 10-frame Langstroth deep hive boxes (Dadant & Sons, Inc., Hamilton, IL, USA) with plastic comb foundations. Before the experiment, the colonies uniformly occupied six to eight brood combs. The bees in the experimental colonies occupied all of the spaces between the combs and spaces between the hive wall and lateral comb. The Varroa population was estimated by counting the natural Varroa mortality and by performing the sugar shake diagnostic method prior to and after the specific treatments. The corrugated plastic bottom boards (Dadant & Sons, Inc., Hamilton, IL, USA) were inserted to capture dead Varroa for calculating the mite mortality. Wire screens were installed above the boards to prevent the bees from coming into contact with the dead mites and pesticide contaminated debris. On 21 occasions, from 31 May to 21 September, at 3 to 4 day intervals, the dead mites were counted and the bottom boards were cleaned, Figure 2. The levels of the natural mite ‘drop-down’ were recorded on the first 7 of these 21 occasions (sampling dates), before the treatment applications. The number of Varroa that were not killed by the treatments was also obtained by counting the Varroa drop-down during the final CheckMite^®^ treatment period, between 18 August and 21 September, Figure 2. The percentage of the Varroa mites that were killed (PVK) by the first treatment protocol, using Apiguard^®^, Apistan^®^, Apivar^®^, and HopGuard^®^, was calculated using the following equation: PVK T1 = (T1/(T1 + T2) × 100)%, where (T1) is the number of Varroa killed by the first treatments and (T2) is those killed by the second CheckMite^®^ treatment [6]. T1 denotes the total number of mites that dropped after the first acaricidal application, while T2 denotes the number of varroa that were collected after the final CheckMite^®^ treatment on 18 August. The treatment efficacies were estimated by comparing the number of Varroa that were killed before, and then again after the application of the treatment.

The counts continued on 14 sampling dates post-application, from 19 July to 21 September, Figure 2. All of the colonies were similarly managed, until 19 July, when they were randomly assigned to five experimental groups, each group contained four colonies and each group was assigned to one of the five treatments, namely: (1) Apiguard^®^; (2) Apistan^®^; (3) Apivar^®^; (4) HopGuard^®^; and (5) untreated-control. All of the treatments were applied to the colonies according to their respective manufacturers’ instructions. Group 1 colonies each received one tray, which contained 50 g of Apiguard^®^ gel that was placed on the top bars of the frames. The trays were replaced on 31 July and were removed from the colonies on 18 August. Group 2 colonies each received two strips of Apistan^®^. Group 3 colonies each received two strips of Apivar^®^. Group 4 colonies each received two strips of HopGuard^®^. Group 5 colonies were left untreated and served as the control group.

All of the Acaricide strips and Apiguard^®^ trays were removed from all of the colonies on 18 August. To evaluate the efficiency of each treatment and to quantify the Varroa mite population in each colony, a final standard CheckMite^®^ treatment (2 strips/colony) was applied in each experimental colony on the 18 August, Figure 2. The Varroa drops that were as a result of this final treatment, were regularly counted on the hive bottom boards, until 21 September. To determine the relative infestation levels (number of Varroa mites per bee) in the experimental colonies, a sugar shake test [23] was conducted on the adult bees. For this test, approximately 300–400 worker honey bees were brushed into 900 mL glass jars with a 3.1-mm mesh screen in their lids. Two tablespoons of powdered sugar were added through the screen and the jars were rolled, in order to distribute the sugar evenly over the bees. After 1 min, the jar and the honey bees were vigorously shaken over a white paper plate for about 4 min, and the dislodged Varroa were then counted. This method involved sampling the adult bees from the middle brood frames at three portions of the experiment, namely: (1) at pre-treatment on 19 July; (2) post-treatment, right after removing the four acaricide treatments on 18 August; and (3) the final sugar shake, which occurred after the removal of the CheckMite^®^ strips on 21 September, Figure 2. The percentage of the Varroa infestation was calculated based on the number of the Varroa mites that were found on or removed from 100 worker bees. During the sugar shakes, the following outside temperatures (T) and relative humidity values (RH) were recorded: 19 July: T = 32 °C, RH = 61%; 18 August: T = 33 °C, RH = 56%; and 21 September: T = 30 °C, RH = 53%. 

### 2.3. Data and Statistical Analyses

The Varroa counts in the hive experiment were log_10_-transformed so as to improve the normality and to better visualize the Varroa population trends within the honey bee colonies. All of the statistical analyses and figure generation were carried out in the R environment [24]. The two-way ANOVA (analysis of variance, α = 0.05) and Tukey HSD test identified significant differences among the treatment groups and dates within the treatment group. The bars in the figures represent the ± standard error (±SE). The sugar shake differences between the Varroa adult bee infestation before and after the acaricidal treatment, as well as after the final CheckMite^®^ treatment, were analyzed and compared with the relative cumulative Varroa mortalities after the acaricides treatments, which was followed by the final CheckMite^®^ application. 

## 3. Results

### 3.1. Laboratory Toxicity Experiment 

**Figure 3:**
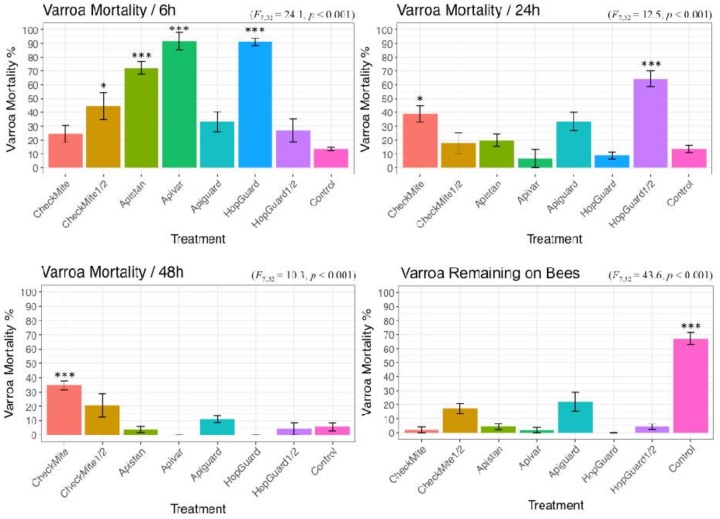
Varroa mite mortality recorded in each caged-bee group at intervals of 6, 24, and 48 h. Groups were exposed to a CheckMite^®^ 1 dose, CheckMite^®^ ½ dose, Apistan^®^, Apivar^®^, Apiguard^®^, HopGuard^®^ 1 dose, HopGuard^®^ ½ dose, or left untreated (control group). The term ‘remaining’ denotes the percentage of Varroa that survived after 48 h of exposure. Asterisks show ANOVA significance levels for pairwise comparisons between the control and each acaricidal treatment and they are as follows: *p* < 0.05 * and *p* < 0.001 ***. Bars denote the mean Varroa mortality (%) ± 1 standard error (SE).

**Figure 4:**
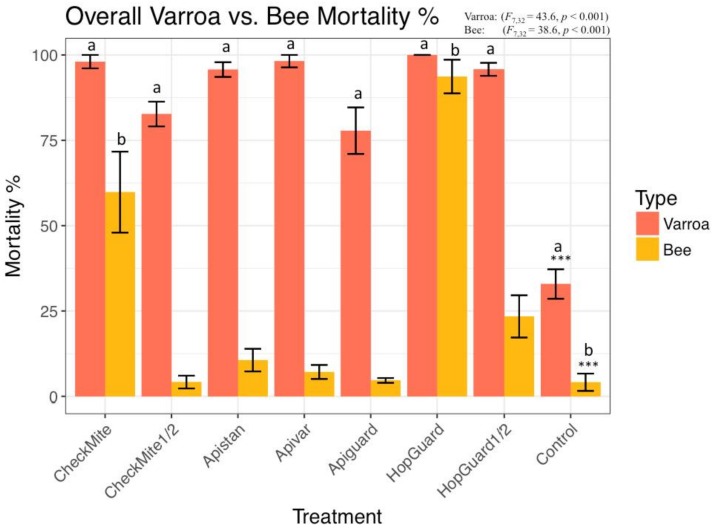
Total mortality for Varroa mites and their caged host bees after 48 h of exposure to 1 dose of CheckMite^®^, ½ dose of CheckMite^®^, Apistan^®^, Apivar^®^, Apiguard^®^, 1 dose of HopGuard^®^, ½ dose of HopGuard^®^, and no acaricide (control group). Same letters indicate significant differences between a treatment and control at α = 0.05. The only significant differences that were detected occurred at *p* < 0.001 (***). Bars denote mean bee and mite mortality (%) ± 1 standard error (SE).

The highest Varroa mortality occurred for the Apivar^®^ (91.51% ± 6.45), HopGuard^®^ (91.11% ± 2.63), and a single Apistan^®^ dose (72.22% ± 4.51), within the first six hours of exposure. The Varroa mortality continue during 24 and 48 h of exposure. The highest cumulative Varroa mortality during the 48 h of exposure was recorded in the Apivar^®^ (98.18% ± 1.8) and CheckMite^®^ 1 (98.03% ± 1.9) treatment groups. The CheckMite^®^ 1 also induced a high bee mortality at the level of 59.82% ± 11.86, in comparison with the CheckMite^®^ ½ dose, which induced a 4.18% (±1.8) bee mortality. The Apistan caused a 95.72% (±2.1) Varroa moratality. The HopGuard^®^ 1 dose simultaneously induced a 100% Varroa mortality and a 93.68% ± 4.92 bee mortality. On the other hand, the HopGuard^®^ ½ dose induced a 95.79% (±1.9) Varroa mortality and a 23.41% (±6.1) bee mortality. The Varroa and bees in cages that were not exposed to an acaricide during the duration of the experiment also died at the levels of 32.90% (±4.3) and 4.12% (±2.5), respectively. Significant differences in the Varroa mortalities were found between the treatment groups (F = 43.67; df = 7; *p* < 0.01; Figure 3 and Figure 4).

The mortality in the caged-bees varied significantly among the treatments (F = 38.63 df = 7; and *p* < 0.01). The highest bee mortality was observed in the cages that were treated with HopGuard^®^ 1 dose and CheckMite^®^ 1 dose. The lowest bee mortalities occurred in the colonies that were treated with the CheckMite^®^ ½ dose 4.18% (±1.8), Apistan^®^ 10.61% (±3.3), and Apivar^®^ 7.13% (±2.0) (Figure 4).

### 3.2. Field Experiment

**Figure 5:**
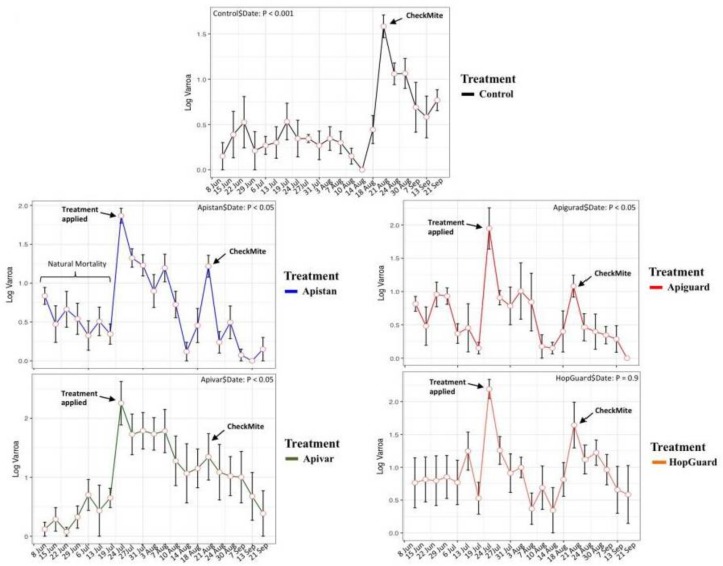
Overview of the Varroa population dynamics and the Varroa mortalities after specific acaricide treatment within colonies of the four treatment groups and control. Acaricidal treatments were applied on 19 July in all of the groups except the control, and a final CheckMite^®^ treatment was applied in all of the groups on 18 August, so as to kill the remaining mites in a hive. The period between 31 May and 19 July is, for all of the groups, a monitoring period to assess the natural Varroa mortality, in which no acaricides were applied.

**Figure 6:**
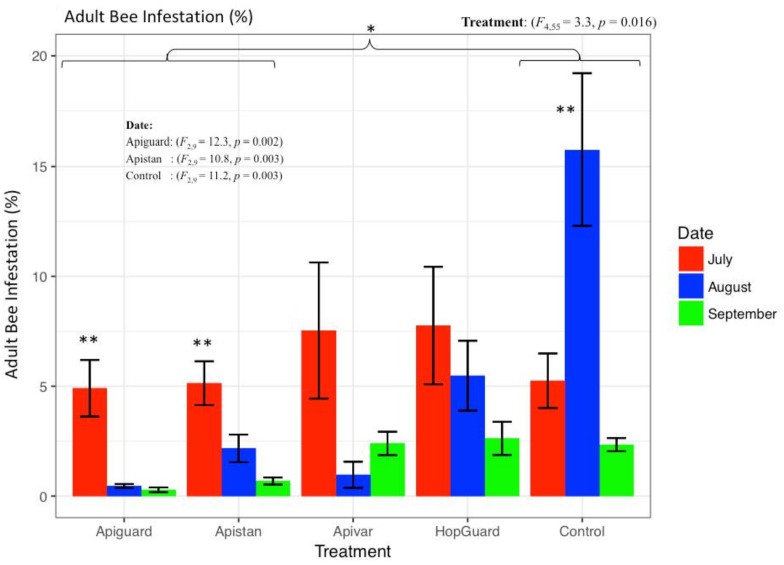
Relative adult bees infestations in three time periods, determined using the sugar shake method. Adult bee infestations before the application of four acaricides, Apiguard^®^, Apistan^®^, Apivar^®^, and HopGuard^®^ (period before treatments from 31 May–19 July, shown as July in figure) and after treatments (19 July–18 August, shown as August and September in figure, respectively). Denote that colonies of the control group received the acaricide treatment on 18 August and therefore a high Varroa infestation was recorded, as August testing, just before CheckMite^®^ treatment. CheckMite^®^ was applied as a final treatment to kill any remaining Varroa mites in all of the colonies. Varroa infestation levels (% ± SE) were calculated based on the relative number of mites remaining on the adult bees in each colony. The horizontal brackets indicate the mean comparison between the control and Apiguard^®^ and Apistan^®^ as a single treatment; * *p* < 0.05; ** *p* < 0.005.

Before the treatment on 19 July, the 20 honey bee colonies had exhibited a relatively uniform rate of natural Varroa mortality, which averaged 0.89 ± 1.06 mites per day (F = 0.90, df = 4, *p* = 0.48). During the pre-treatment period, 17.25% ± 4.05 of the Varroa fell onto the bottom board inserts. Three of the four acaricides that were used in the honey bee colonies (Apiguard^®^, Apistan^®^, and Apivar^®^) induced steep daily and monthly declines in the Varroa mite density within the honey bee colonies (*p* < 0.05, Figure 5 and Figure 6). On the other hand, the HopGuard^®^ differed from the other treatments, in that it had a more uniform impact on the Varroa mortality throughout the treatment period (*p* = 0.9, Figure 5). The Varroa mortalities in the experimental groups were increased after each specific acaricide treatment. This was also demonstrated in the control colonies after having received a single CheckMite treatment, as shown in Figure 5. The overall relative arroa mortality showed no significant differences among the colonies that were exposed to the four different acaricides that were used in our study. However, the significant differences were recorded between all of the treatment groups (F = 25.8, df = 5, *p* < 0.001) and both the Varroa mortality in the control, untreated colonies, and the natural Varroa mortality (pretreatment), with no differences being recorded between the last two groups.

**Figure 7:**
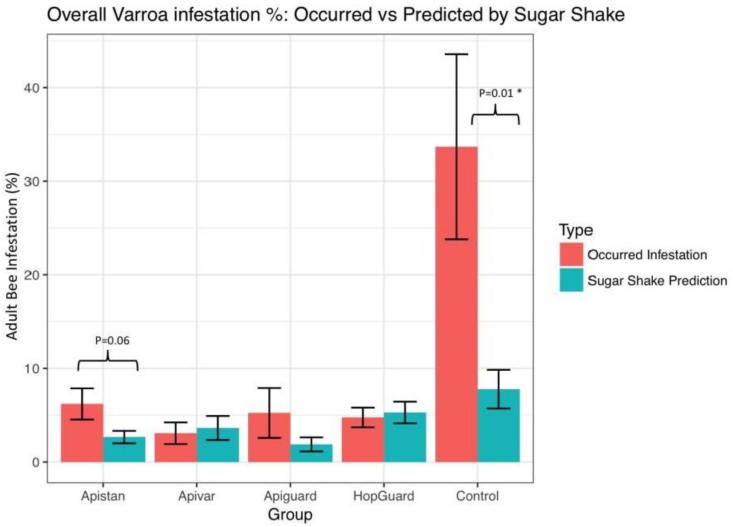
Percentage of adult bees infested by Varroa mites, detected by sugar shake test, during the pre-treatment period (July), after four treatment protocols (Apiguard^®^, Apistan^®^, Apivar^®^, and HopGuard^®^) (August) and final treatment of CheckMite^®^ (September). Bars represent mean ± 1 SE. ANOVA significant levels are as follows: *p* < 0.05 *. The horizontal brackets indicate significant differences between the actual and predicted levels of Varroa mite infestation.

**Figure 8:**
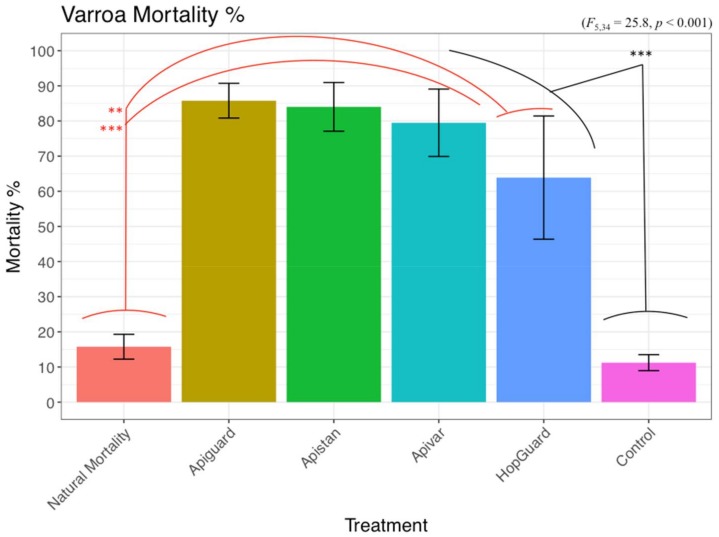
Final actual relative Varroa mortality levels derived from the sugar shake method, compared with actual Varroa infestation calculated from bottom board counts. The ‘actual varroa infestation’ was calculated based on the total number of Varroa mites, counted from each colony at the end of the experiment, after the removal of the CheckMite^®^ strips. Denote that Varroa mortality levels represent the calculated relative efficacies of acaricides applied to the honey bee colonies in the field.

**Figure 9:**
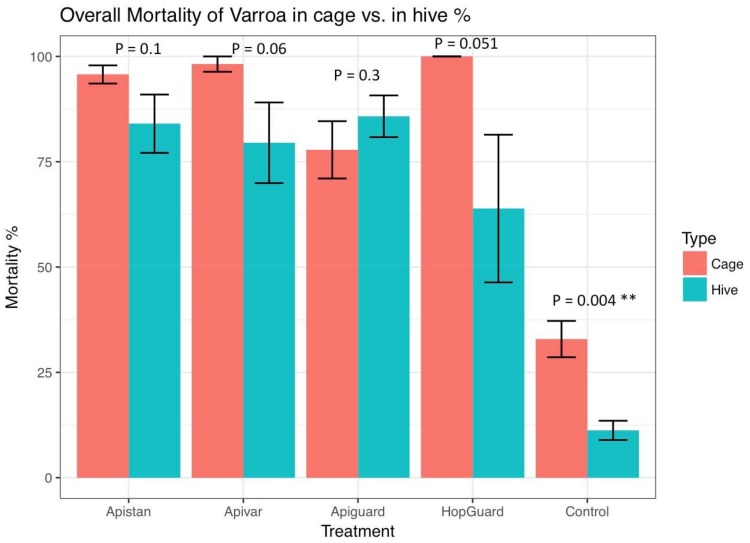
Comparative relative efficacy of Apiguard^®^, Apistan^®^, Apivar^®^, and HopGuard^®^ applied to both caged bees and honey bee colonies, between 19 July and 18 August. All Varroa mites from the caged bees were counted after 48 h of exposure and after the final CheckMite^®^ application in colonies. Bars indicate mean ± 1 standard error (SE). ANOVA significant level is *p* < 0.01 **.

The Varroa infestation of adult bees was estimated at 6.12% ± 1.93% in the pre-treatment period, with no significant differences in the Varroa among the colonies. Although the control colonies experienced a slight seasonal decline in Varroa numbers, the sugar shake sampling method showed a much more profound reduction in the mite infestations of the colonies that were treated with Apiguard^®^ and Apistan^®^ (*p* < 0.05, Figure 5, Figure 6 and Figure 7). The adult bees’ infestation, which was detected with the sugar shake test prior to the Apiguard, Apistan, Apivar, and HopGuard treatment, was 4.90% (±1.2), 5.13% (±0.9), 7.53% (±3.0), and 7.76% (±2.6), respectively; while the adult bee infestation after the specific acaricides treatments was 0.45% (±0.09), 2.16% (±0.62), 0.97% (±0.5), and 5.47% (±1.5), respectively. A significantly increased adult bee infestation during the treatment period was recorded in the control, untreated colonies. From the initial 5.25% (±1.2), the adult bee infestation increased to a 15.75% (±3.4) infestation. The CheckMite application induced reduction in the adult bee infestation was detected by the sugar shake test in September, at a level of 2.34% (±0.3) (Figure 6). The sugar shake indicated a significantly higher Varroa infestation in August (*p* < 0.01) for the control colonies. The overall ‘sugar shake prediction’ estimation of the Varroa mite load that was obtained by performing a sugar shake test in the honey bee colonies, came in full agreement with the ‘occurred Varroa infestation’, in terms of the killed varroa mites after the CheckMite^®^ application, which was obtained by counting the killed Varroa mites on the bottom boards. No significant differences in the dead Varroa were found between the sugar shake prediction and the occurred Varroa infestation, which were detected on bottom boards, except for the control group. During the CheckMite^®^ strips application to the treatment groups, the relative efficacies for Apiguard^®^, Apistan^®^, Apivar^®^, or HopGuard^®^ were recorded at the levels of 86%, 84%, 79%, and 64%, respectively. During the same period in the control, the untreated colonies’ natural Varroa mortality remained at the level of 11% (Figure 8). The comparative relative efficacies of the acaricide treatments that were conducted on the caged bees versus the bees in the experimental colonies in the field were found to be the same (Figure 9).

## 4. Discussion

The five commercial acaricides that were used in our experiments demonstrated the variable levels of the Varroa mite mortalities, as shown in the laboratory and field tests. However, the mite knockdown did vary among the acaricides during the first 48 days post-treatment. Apivar^®^, Apistan^®^, and a full dose of HopGuard^®^ killed most of the Varroa within the first six hours of exposure, whereas the residual activity diminished rapidly past 6 h. In contrast, CheckMite^®^ and Apiguard^®^, for 48 h, displayed more uniform and persistent acaricidal activity against the mites on the caged bees. It wass important to note that, even after 48 h of exposure, none of acaricides eradicated all of the Varroa mites on the caged honey bees, despite the naturally high mortality among the mites. In fact, after 48 h, ~33% of the Varroa mites died in the control cages. 

Coumaphos in CheckMite^®^ originally acted systemically, whereby the bees consumed small quantities and spread the acaricide trophalactically. However, when bees’ bodies had direct contact with the coumaphos-impregnated strips, more of the acaricide was distributed dermally among the nestmates [25,26], thereby increasing the chances of the worker bees receiving an acute lethal dose (LD_50_ = 3 μg to 6 μg per bee). Within a colony, the individual bees that were in direct contact with the CheckMite^®^ strips containing ~1300 mg of the active ingredient, could receive much higher coumaphos doses. Acaricidal toxicity was more pronounced when the caged host bees were kept in close contact with the treatment strips. Likewise, the bees in the highly congested hives with less living space might have similarly and more frequently had with the contact acaricidal strips. However, a bees’ age, social interactions among nestmates and brood, food stores, and other factors could, to some extent, limit the honey bees’ exposure or sensitivity to coumaphos and other neurotoxic acaricides [25]. The additional bee exposure could have occurred as the workers walked on and handled the coumaphos-contaminated wax, which could have contained the coumaphos residues from 1.0 μg/kg to 919 mg/kg [27]. Other routes of exposure included the bees feeding on honey or handling propolis [9,28,29,30]. Clearly, direct contact with the acaricidal strips, which contained a maximum dose of 83 mg a.i. coumaphos, could have been acutely fatal to the honey bees, while half of this dose was largely benign to the workers. Lowering the coumaphos dose by half would have helped to prevent the unacceptable bee losses, while the active ingredient remained as effective as the full dose, for killing the Varroa. Thus, at appropriate rates, the coumaphos could be regarded as safe, or at most, mildly toxic to honey bees [31]. Our studies showed that some acute bee mortality could occur in both the caged bees and field colonies [32], but the losses could be mitigated with careful mite monitoring and strip dosing. Apistan^®^ was found to be highly effective against the Varroa mites in the caged, as well as in the honey bee colonies experiments. It seemed that the newly established colonies that had not been previously treated with tau-fluvalinate, carried Varroa that were sensitive to this active ingredient. It was therefore important to test the acaricide efficacy against the Varroa prior to the application to the honey bee colonies in the field, as resistance to Apistan^®^ might have been present in the managed honey bee populations [33]

Similar to the coumaphos toxicity to honey bees, mortality was observed in the caged bees that were exposed to both rates of HopGuard^®^, 532 mg per strip and 1064 mg per strip, particularly the latter. This was expected, given that a HopGuard^®^ rate of 150 μg/bee was deemed as safe for honey bees, and induced a mortality of 5% [21]. In our experiment, 23 % of the bees died after exposure to 532 mg of beta acids on the HopGuard^®^ (1/2) strip. The bee mortalities that were observed after the application of CheckMite^®^, full dose, or HopGuard^®^, full dose, to the caged bees, might have varied in the field, because of the changes in the interior hive environment or hive demographics. Further research would be needed in order to assess the efficacies of the various acaricides throughout the reproductive cycles of the Varroa mites and their bee hosts. Likewise, we should also identify any possible interactions that have occurred between the acaricides and the climate (e.g., subarctic, alpine, temperate, subtropical, and tropical) in which the colonies are managed. 

The bee mortality that was observed in the laboratory tests was far less or absent in the honey bee colonies, except for the HopGuard^®^ 1 dose and HopGuard^®^ ½ dose that was applied in the cages test, which killed between 23% and 94% of our bees, and was known to be highly toxic to both mites and honey bees [21]. Nevertheless, the HopGuard was an efficacious acaricide that killed >90% of the mites, and it remained equally efficacious after halving the full test dose. This reduced rate of the HopGuard kept the bee mortality to an acceptable level, just slightly above the control bee mortality. Therefore, we propose that acaricide treatments, including HopGuard, that are applied at doses that cause >70% mite mortality, with less than 30% bee kill, should be considered as mite selective and acceptable for mite management [16]. 

Apistan^®^, Apivar^®^ strips, and Apiguard^®^ gel were relatively safe to adult bees, as we recorded approximately 10%, 7%, and 5% bee mortality, respectively, in our cages test. A low bee mortality at the level of 4% was also recorded when we used CheckMite^®^ ½ dose. It was interesting that, by doubling the coumaphos dose, the bee mortality was drastically increased by up to 60%. On the other hand, the acaricides Apiguard^®^ and CheckMite^®^ ½ dose were less effective at killing the Varroa, at least when compared with Apistan^®^, Apivar^®^, or HopGuard^®^ ½ dose ,in which bee mortality was on tolerable levels in the cage experiments. The Varroa mortality increased after Apiguard^®^, Apistan^®^, Apivar^®^, or HopGuard^®^ were introduced into field colonies that contained brood. The relative efficacy of the Varroa control in the honey bee colonies, using the previously mentioned acaricides, Apiguard^®^, Apistan^®^, Apivar^®^, or HopGuard^®^, which were performed between 19 July and 21 September, as were determined by using the sugar shake method and calculating the Varroa mortalities that were detected on the hives’ bottom boards in our experiment was 86%, 84%, 79%, and 64%, respectively. The natural Varroa mortality during the pre-treatment period of 49 days was less than one mite per day, and during the pretreatment period. The natural mite mortality in the control (untreated) colonies rose to an average of 3.7 mites/day, as detected on the bottom boards. The final CheckMite^®^ treatment that was completed on the previous control colonies, ensured that these colonies survived, particularly given that the unchecked Varroa populations continued to rise before the CheckMite^®^ strips were installed. It was therefore evident that the sugar shake test revealed that approximately 16% of the adult bees in the untreated control colonies were infested prior to the CheckMite^®^ application. After treatment, the adult bee infestation was reduced to 2%. 

Amitraz, as the active substance (formamidine) in the Apivar^®^ product, was an effective acaricide, based on our laboratory tests with the caged bees that were infested with Varroa. It was one of the first chemicals that was tested in 1979 for the control of the Varroa mite in the managed honey bee colonies [34]. In previous years, the reports of the variable efficacies of Amitraz, in order to control the Varroa, indicated that the mite populations were becoming resistant [35,36,37]. Mite resistance to Amitraz around the globe varied considerably from 0% to 96%, depending on the acaricide management that was adopted [38,39]. Our populations of Varroa mites appeared highly susceptible to Amitraz (Apivar^®^) and this product was highly effective for controlling the Varroa mites in both the cage and field experiments, with mean mortalities of ~98% and ~79%, respectively. A lower efficacy was established in the colonies after using the coumaphos treatment and counting the dead Varroa that were on the bottom boards, in comparison to the estimated efficacy using the sugar shake, and the survived Varroa were counted on the adult bees. Our efficacy values for the Apivar that was obtained in the cages test concurred with those that were derived in different studies, namely: 78.8–87.3% [38], 98.4–99.5%, [40], and ≤60.1% [41]. 

Different degrees of efficacy had been established for Apiguard^®^. In one experiment, the mortality rate of the Varroa mite after the Apiguard^®^ treatments was 86%, whereas, in others, the natural mortality rate in the control colonies was approximately 23% [19]. It was also interesting that the 46% efficacy of the Apiguard^®^ in the colonies with brood in the continental climatic conditions [20] was lower than our current findings. Different climatic and geographic conditions, as well as hive management systems may affect Apiguard^®^ efficacy against varroa [2,42]. Perhaps, Apiguard^®^ efficacy, increased when they were applied to the colonies that were managed in a subtropical climate. It was noteworthy that the Apiguard^®^ treatments that were conducted in autumn had little to no negative effect on the early spring bee populations [7].

The results of this study gave an indication of the limited effect of HopGuard^®^ as a long-term or persistent organic treatment in honey bee colonies, in comparison to the other acaricides that were tested. The effectiveness of HopGuard^®^ in colonies with brood lasted about seven days. Therefore, to extend the residual activity of HopGuard^®^ in the colonies with brood, three consecutive HopGuard^®^ treatments should have been applied [43]. This organic treatment thus effectively contributed to the Varroa reduction during the season and, coupled with other control treatment applications during late summer and autumn, could reduce an infestation to an almost negligible level, which was confirmed by the sugar shake test that was completed in September, which showed the low infestation levels in the colonies. The higher efficacy in killing the Varroa mites occurred after late summer. For example, the September treatments induced a higher mite mortality when compared with the earlier treatments, because of a larger proportion of more susceptible phoretic Varroa on adult bees [44]. We also confirmed the high efficacy of HopGuard^®^ against the Varroa mites on adult caged bees. Therefore, this product could be applied to colonies with or without brood [43]. The efficacy of the treatments could have been underestimated because of the reproduction of the surviving Varroa and the mite re-invasions of the experimental colonies [45]. Alternatively, the Varroa mortalities might have have been overestimated when the CheckMite^®^ was applied as a final treatment in the colonies, which was less than 100% effective in late September. Throughout the season, all of the treated and control colonies developed normally and remained strong without any mass deaths occurring among the worker bees or queens [46,47]. It was noteworthy that the efficacy of controlling the Varroa in cages corresponded to the efficacy of the Varroa control in the colonies. 

The rapid growth of the mite populations in the untreated control colonies showed that the low mite populations in the spring and early summer did not always remain low, even when the honey bee populations began to decline in the fall and winter. Therefore, sampling bees throughout the season, to count mites, or performing the sugar shake test, to remove the mites for counting, might be required in order to ensure the seasonal control of the Varroa with ≥70% efficacy [23,48]. The sugar shake test, which was as effective at counting the Varroa on the hive bottoms, was a more convenient test for monitoring the Varroa populations and for assessing the acaricide effectiveness. An accurate diagnosis ensured a good projection of a colony’s real infestation level, and assisted in determining the potential mite population growth in the colonies. A powdered sugar shake, followed by an acaricide treatment, organic or conventional, could be used to diagnose and treat Varroa promptly. 

## 5. Conclusions

Our results show that various acaricides are variably effective for Varroa suppression when the mite populations are rising and brood is present. Hence, colonies that are treated with HopGuard^®^ in summer will require another treatment in autumn, so as to prevent a Varroa resurgence. When there are smaller host broods within the honey bee colonies, both the conventional and organic acaricides are effective Varroa controls. However, the control must be preceded by an accurate Varroa diagnoses, in order to maintain the mite levels below an economic threshold that is needed for the colony’s survival.
